# Screening for clusters of charge in human virus proteomes

**DOI:** 10.1186/s12864-016-3086-3

**Published:** 2016-10-17

**Authors:** Najla Kharrat, Sabrine Belmabrouk, Rania Abdelhedi, Riadh Benmarzoug, Mourad Assidi, Mohammed H. Al Qahtani, Ahmed Rebai

**Affiliations:** 1 Centre of Biotechnology of Sfax, Laboratory of Molecular and Cellular Screening Processes, Bioinformatics Group, PO. Box:1177, 3018 Sfax, Tunisia; 2 Center of Excellence in Genomic Medicine Research, King Abdulaziz University, Jeddah, Saudi Arabia; 3 Center of Innovation in Personalized Medicine, King Abdulaziz University, Jeddah, Saudi Arabia

**Keywords:** Cluster charge, Proteomes, Virus, Human, Screening

## Abstract

**Background:**

The identification of charge clusters (runs of charged residues) in proteins and their mapping within the protein structure sequence is an important step toward a comprehensive analysis of how these particular motifs mediate, via electrostatic interactions, various molecular processes such as protein sorting, translocation, docking, orientation and binding to DNA and to other proteins. Few algorithms that specifically identify these charge clusters have been designed and described in the literature. In this study, 197 distinctive human viral proteomes were screened for the occurrence of charge clusters (CC) using a new computational approach.

**Results:**

Three hundred and seventy three CC have been identified within the 2549 viral protein sequences screened. The number of protein sequences that are CC-free is 2176 (85.3 %) while 150 and 180 proteins contained positive charge (PCC) and negative charge clusters (NCC), respectively. The NCCs (211 detected) were more prevalent than PCC (162). PCC-containing proteins are significantly longer than those having NCCs (*p* = 2.10^-16^). The most prevalent virus families having PCC and NCC were *Herpesviridae* followed by *Papillomaviridae*. However, the single-strand RNA group has in average three times more NCC than PCC. According to the functional domain classification, a significant difference in distribution was observed between PCC and NCC (*p* = 2. 10^−8^) with the occurrence of NCCs being more frequent in C-terminal region while PCC more often fall within functional domains. Only 29 proteins sequences contained both NCC and PCC. Moreover, 101 NCC were conserved in 84 proteins while only 62 PCC were conserved in 60 protein sequences. To understand the mechanism by which the membrane translocation functionalities are embedded in viral proteins, we screened our PCC for sequences corresponding to cell-penetrating peptides (CPPs) using two online databases: *CellPPd* and *CPPpred*. We found that all our PCCs, having length varying from 7 to 30 amino-acids were predicted as CPPs. Experimental validation is required to improve our understanding of the role of these PCCs in viral infection process.

**Conclusions:**

Screening distinctive cluster charges in viral proteomes suggested a functional role of these protein regions and might provide potential clues to improve the current understanding of viral diseases in order to tailor better preventive and therapeutic approaches.

**Electronic supplementary material:**

The online version of this article (doi:10.1186/s12864-016-3086-3) contains supplementary material, which is available to authorized users.

## Background

In protein sequences, compositionally biased (CB) regions are segments whose amino acids’ composition is significantly different from the average amino acid usage of the proteome [1, 2]. CBs are abundant in proteomes and significantly diverge from one protein family to another depending on function [3]. These regions often exist in intrinsically disordered domains together with ordered domains in the same proteins and are, most of the time, involved in regulatory functions [4, 5]. The CB regions are found in all the three domains of life (Bacteria, Archaea and Eukarya) [6–9] and has also been reported in viral genomes [10]. Ekman et al. [11] reported that CB are contained in highly connected “hub” proteins compared to non-hub proteins. Few studies have investigated protein regions with biased composition in positive (Lysine, Arginine) or negative amino acid (Aspartic acid, Glutamic acid) [12–15]. These clusters of charged residues are often grouped, producing hence local concentrations of charge within CB, called charge clusters. These regions are most of the time present at the surface of the tertiary or quaternary structure of proteins and contribute to their stability, folding and/or activation as have been supposedly suggested for some proteins families [16]. They have been described for the first time in association with functional domains of cellular transcription factors by Brendel and Karlin [17]. They are also thought to facilitate intramolecular folding and cooperative protein-protein and protein-nucleic acid interactions. Charge clusters, named also runs of charge residues, appear to play important roles in protein transport, localization, and function(s) regulatory [16, 18–20]. In fact, the flexible nature of regions lacking well-defined folding structures may be responsible for their versatile binding within different targets [21]. For example positive charge clusters (PCCs) are generally associated with signal transduction in the cytoplasm and act as linkers binding within transmembrane proteins [17, 22]. On the other side, negative charge clusters (NCCs) are involved in cation coordination (calcium, magnesium or zinc ions) [22]. Moreover, the presence of both positive and negative charge clusters in the same protein sequence, named mixed charge clusters, is thought to contribute in stabilizing and facilitating quaternary structure formation [23].

The identification of charge clusters and their mapping within protein sequences is of great interest. In fact, it might bring new insights into the mode of interaction between these regions and other regions in target proteins and provide a better understanding of several biological and pathological processes. Only few algorithms dedicated to the identification of these clusters have been described in the literature [14, 15] but, none of them allowed specific identification of CC in proteins. We recently developed a tool called “Finding Charge Cluster in Protein sequences” (FCCP) and reported its efficient use in detecting CC in eukaryotic proteomes [24]. In this study we identified linear and disjoint CCs within protein sequences and get accurate information about the number and position of CCs in proteins of different eukaryotic proteomes. Here we investigate some virus proteomes that have not been addressed before. The only reported work was related to low complexity region as support for source of variability in viral proteins [10]. Furthermore, Blaisdell and Karlin [25] is the only published screening of distinctive cluster charge in Epstein-Barr virus proteome. In this study, a proteome-wide scan of linear CCs in 197 human viruses was performed to identify significant CCs in viral proteins and discuss their potential functional properties.

## Results

### Description of studied proteomes

**Fig. 1:**
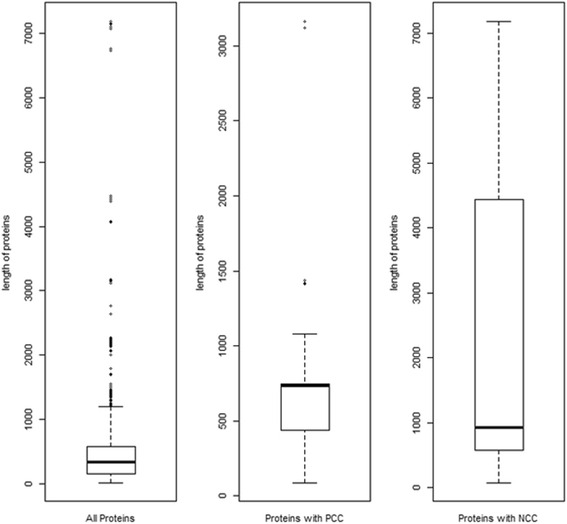
The distribution of the length of all studied protein sequences

In this study, the biggest proteome was that of the Human cytomegalovirus (strain AD169) which contained 191 proteins and the smallest one contained just a single protein and this was the case of 23 viruses (1 Eechovirus, 9 Hepatitis, 3 Parechovirus, 3 Enterovirus, 7 Rhinovirus). The distribution of the length of all studied protein sequences is shown in Fig. 1. The average length was 475.4 ± 571.66 amino acids (aa) (Table 1).

**Table 1:** Distribution of clusters charge in human virus

	Average length (aa)	Minimal length	Maximum length	Average of NCC number	Average of PCC number
Positive cluster charge	521.72 ± 449.20	89	3164		20 ± 9.03
Negative cluster charge	1264.04 ± 1789.90	83	7182	24.47 ± 11.97	
All proteines	475.4 ± 571.66	25	7182	24.47 ± 11.97	20 ± 9.03

Mean ± Standard deviation

*NCC* Negative Cluster Charge, *PCC* Positive Cluster Charge

No positive charge clusters (PCC) were detected in the single-protein viruses and the rate of CC-free proteins varied from 94 to 92 %, respectively for PCC and NCC. The difference between the average length of protein sequences with and without charge clusters is significant (*p* = 4.10^−14^ and *p* = 2. 10^−16^ for PCC and NCC respectively) whatever the cluster type considered. Regarding the mean length of proteins sequences, it was significantly different between PCC and NCC (*p* = 2. 10^−16^). NCCs were more prevalent than PCCs, with 211 NCC against 162 PCC detected in the overall 2549 protein sequences.

### Occurrence of positive charge clusters

One hundred sixty two (162) PCC were identified in 148 protein sequences. Among these, only 15 proteins contained two PCC while all others had just only one. The PCC-containing proteins belong to 10 virus families (Anelloviridae, Astroviridae, Adenoviridae, Herpesviridae, Retroviridae, Paramyxoviridae, Reoviridae, Papillomaviridae,Hepadnaviridae, Picobirnaviridae) and to 5 different virus groups (dsDNA viruses, ssRNA viruses, dsRNA viruses, ssDNA viruses, retro transcribing virus) (Additional files 1 and 2).

The highest prevalence of positive charge clusters was found in Herpesviridae family (37 % of PCC) followed by Papillomaviridae (22 %). Though, the poorest proteomes in charge clusters were Picobirnaviridae, Paramyxoviridae and Hepadnaviridae (1 %). We noted that the PCC were more prevalent in dsDNA (63 %) than ssRNA or dsRNA (7 %) groups (Additional file 2).

The average size of PCC-containing proteins was 522 ± 450 aa and 75 % of these proteins are smaller than 546 aa.

The analysis of variance revealed significant differences for the length of protein sequences between groups of virus (*p* = 3. 10^−5^) and families (*p* = 10^−5^). The size of PCCs varied from6 to 61 aa. The average size was 21 ± 9 aa. A significant difference for the CC size was noted between group of virus (*p* < 10^−16^) and families (*p* < 10^−16^).

The shortest PCC was found in Epstein - Barr virus and was conserved within 3 strains, for the same protein (Latent membrane protein 2). This latter maintains EBV latent infection of B-lymphocyte, by preventing lytic reactivation of the virus in response to surface immunoglobulin (sIg) cross-linking. It acts as a dominant negative inhibitor of the sIg-associated protein tyrosine kinases, LYN and SYK. It also blocks translocation of the B-cellantigen receptor (BCR) into lipid rafts, preventing the subsequent signaling and accelerated internalization of the BCR upon BCR cross-linking. It serves as a molecular scaffold to recruit SYK, LYN and E3 protein-ubiquitin ligases, such as ITCH and NEDD4L, leading to ubiquitination and potential degradation of both tyrosines kinases. It also possesses a constitutive signaling activity in non-transformed cells, inducing bypass of normal B lymphocyte developmental checkpoints allowing immunoglobulin-negative cells to colonize peripheral lymphoid organs [26].

The longest PCC (61 aa) belongs to unclassified Anelloviridae family and seems to play a role in the self-assembly of the icosahedral capsid. In addition, the PCC (52–54 aa) was detected in capsid protein sequences and particularly within Torque teno virus.

### Mapping of positive charge clusters relative to protein domains

According to Pfam [27] database, the PCCs are more commonly located in the functional domains (97), than in N-terminal (10) or in Interdomain (14) or C-terminal regions (5) The Fisher exact test also showed a significant difference between virus groups according to the functional domain classifications (*p* < 10^−16^ according to *coin* R package) (Additional file 3).

A significant difference in distribution was also observed between PCC and NCC (*p* = 2. 10^−8^) with the occurrence of NCC being more frequent in C-terminal region and of PCC within functional domains (Additional file 3).

### Occurrence of negative charge clusters

The number of NCC detected was 211 in 183 protein sequences. The majority of the proteins (75 %) had only one NCC while 15 proteins have two NCCs, three proteins contained 3 NCC and only two proteins had 4 NCC.

The average size of NCC-containing proteins is 1264 ± 1790 aa and about 75 % of the proteins having NCCs have a size higher than 941 aa. The NCC were screened in 9 virus families (Astroviridae, Herpesviridae, Anelloviridae, Adenoviridae, Retroviridae, Reoviridae, Coronoviridae, Papillomaviridae, Poxviridae).

Similar to PCC, the families with the highest number of NCC in proteome are the Herpesviridae (50 %) followed by Papillomaviridae virus (14 %) (Additional file 4).

The analysis of variance revealed significant differences for the length of protein sequences between virus groups (*p* = 10^−16^) and families (*p* = 10^−16^).

The size of NCCs varies from 7 to 60 aa. The average size was 25 ± 12 aa. A significant difference in NCC length was noted between virus groups (*p* < 10^−16^) and families (*p* < 10^−16^).

While the smallest NCC (7 aa) was detected in an uncharacterized protein RL12 from cytomegalovirus, the largest (60 aa) one was found in Tegument protein UL47 homolog from Varicellovirus. This protein is reported to bind to various RNA transcripts, plays a role in the attenuation of selective viral and cellular mRNA degradation by modulating the activity of host shutoff RNase ORF17/VHS. Possible involvement in the primary envelopment of virions in the perinuclear space, probably by interacting with two nuclear egress proteins ORF24 and ORF27 was also described [28].

**Fig. 2:**
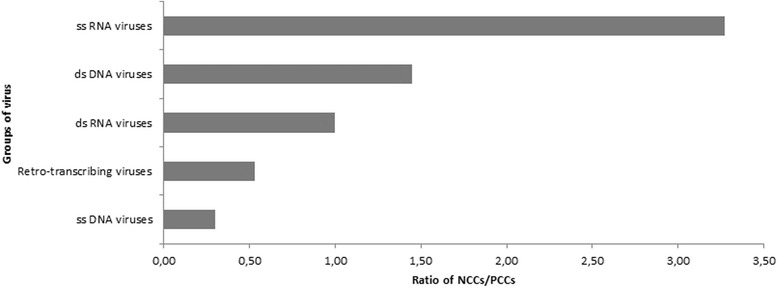
The ratio of NCCs and the number of PCCs in virus groups

There was a significant difference between the distribution of NCC and PCC among virus families and groups (*p* = 6.10^−4^; *p* = 10^−5^). Also, a significant difference of size between NCC and PCC was found (*p* < 10^−4^). In fact, NCCs are in average three times more prevalent than PCCs in ssRNA viruses and this ratio varied among groups (Fig. 2).

Likewise, the NCC were more prevalent in the functional domains (85) than in C-terminal (46), Interdomain (34) and N-terminal regions (29). The Fisher exact test also showed a significant difference between virus groups according to the functional domain classifications (*p* = 10^−16^) (Additional file 3).

### Mixed charge clusters

Only 29 protein sequences contained both type of CCs (called mixed CCs). In fact, these proteins hold exactly one PCC and one NCC, except for one protein (a capsid protein of Torque teno virus TVV4) which has two PCCs and one NCC and is found in unclassified Anelloviridae. It is a protein involved in the self-assembly of icosahedral capsid [29].

The protein sequences with mixed CCs are involved in several functions including self-assembling, cleavage of the polyprotein into functional products, up-regulation of the viral expression and regulation of productive infection.

These mixed charge clusters were present in four families (Anelloviridae, Astroviridae, Herpesviridae Reoviridae) (Additional file 5) and four groups of viruses (dsDNA, dsRNA, ssDNA, ssRNA) (Additional file 6). Both NCC and PCC in proteins having mixed CC, are located in interdomain and in domains at equal rates (Additional file 3). No significant difference in their distribution was found (*p* = 0.12).

### Conserved charge clusters

The conserved PCCs are found between two to eight proteins. In fact, the eight proteins having a conserved PCC are non-structural polyprotein 1AB and 1A; these proteins contained the viral protease participating in the cleavage of the polyprotein into functional products. It contains also the activities necessary for replication of genomic RNA, as well as transcription of subgenomic mRNA [30].

Conserved NCC were found in two to nine proteins. These proteins were involved in genome replication. They also played a crucial rolein the formation of virus factories (viroplasms) when associated with another non structural protein (NSP2). These viroplasms are large inclusions in the cytoplasm where core-like replication intermediates are assembled and RNA replication takes place. They may regulated NSP2-RNA interactions during genome replication, since Non structural protein 5(NSP5) competes with RNA for the same binding site on the NSP2 octamer. These proteins bind to either ssRNA or dsRNA with similar affinities [31].

The number of conserved clusters varied between 62 for the PCC to 101 for the NCC. A difference was illustrated between the distribution of conserved PCC and conserved NCC within virus groups (*p* = 0.007) and families (*p* = 0.032).

Also, a significant difference of size between NCC and PCC was found (*p* < 10^−4^). Both conserved charge clusters were predominatly located in domains within quite difference concerning the NCC conserved that are present also in interdomain (Additional file 7).

### Identification of *De Novo* motifs

In order to identify new motifs present within PCC or NCC screened, we generate two types of FASTA files from the charge clusters detected. These files were submitted separately to MEME (Multiple EM for Motif Elicitation) [32] to discover *De Novo* motifs shared between clusters. Three motifs were found for NCC with significant *E*-*values* (Additional file 8).

Multiple alignments were done, despite the fact that clusters detected have different lengths, but for the two cases of charge clusters. There are three regions conserved between clusters (Additional file 9). These results confirm the presence of conserved motifs shared among clusters. We also used ScanProsite to scan proteins for matches against the PROSITE collection [33] of motifs including user-defined patterns (data not shown). No hits were detected.

## Discussion

We examined the proteomes of a large number of annotated human viruses that are representative of all virus groups, for the occurrence of runs of charged amino acids (referred to as charge clusters or CC) using a specific tool (FCCP) that we have recently developed [24].

In fact, few analyses of CC in proteins have been studied, mostly in cellular genomes. The screening of distinctive CCs in proteins has been reported for the first time 30 years ago in Epstein-Barr virus [12] but no exhaustive study was performed. Indeed, additional studies have been made since that Velasco et al. [10] reported a computer analysis of low complexity regions (LCRs) present in glycoprotein120 (gp120) from HIV1. Their results suggest that LCRs may represent an important source of antigenic variation in HIV-1 population with high number of glycolysation sites. But they did not provide a systematic tool for detecting theses sequence patterns. In fact, since Brendel and Karlin [17], few algorithms have provided a general methodology to assess statistical significance of charge configuration. In their work, Karlin and Brendel [12] provided an application in viral genes for few proteins from Epstein Barr-Virus. Although, they demonstrated also that aggregate significant charge configurations and repeats, found in EBNA1-EBNA4, EBNA5, LYDMA BZLF1, are important functionally for the latent existence and for the initiation of lytic cycle and may be characteristic of these conditions.

In this work, we present the first systematic and proteome-wide analyses of the occurrence of charge clusters in viral proteins. Our tool (FCCP) has already been used to identify significant and disjoint CCs in seven eukaryotic proteomes [24]. In comparison with our previous work [24], we found also that the NCC were more prevalent than PCCs in all studied (eukaryote and prokaryote (data no shown) proteomes. We also demonstrated clearly that human viral proteomes harbor less charged than eukaryotic proteomes, but based on our studies there seems to be no significant difference in the relative frequencies of NCC and PCC in both proteomes.

Brendel et al. [17] have also reported that proteins with charge structures are much more predominant in animal DNA viruses as compared to both animal RNA viruses and prokaryotic viruses. In fact, we noticed that charge clusters were quite frequent in capsid and core proteins, whereas surface (glycol) proteins contain very often NCCS. PCCs or NCCs were abundant in regulatory proteins implicated in transcriptional transactivation and cellular transformation. This contrast might reflect the role of protein charge structures in facilitating competitive virus-host interactions involving the cellular transcription, translation, protein sorting, and transport apparatus [17].

For all proteins screened which structures have been resolved, we found that all CC (PCC and NCC) were detected in intrinsically disordered domains that are also more easily cleaved, confirming similar findings reported by Freire et al. [5]. Indeed, this occurs to facilitate the conversion of separating viral in flaviviruses [34]. Moreover, the multi-functionality in RNA-interacting proteins via disordered domains is frequent even among non enveloped viruses [35]. Despite the fact that the roles of these proteins in genome packaging and RNA chaperoning are to some extent well described and characterized, their roles in the intermediate events, comprising viral fusion and genome delivery into cytoplasm, remain poorly understood.

As reported in the study of Freire et al. [36], we also found that supercharged viral proteins are almost exclusively capsid proteins and per consequence they might confer evolutionary advantage to some virus. So, capsid protein may have genomes transported across membrane among their multiple functionalities [36].

To understand the mechanism by which the membrane translocation functionalities are embedded in viral proteins, we screened our PCC for correspondence to cell-penetrating peptides (CPPs) using two online databases: CellPPd [37] and CPPpred [38]. The results showed that cell-penetrating domains were found even in the other regions of proteins screened (data not shown). In fact, all our PCC encompassing 7–30 amino acids and highly positively charged were predicted as CPPs, for the rest, they cannot be screened, due to the size limit of prediction tools. Experimental investigations are needed to validate these findings.

The abundance of CPP sequences in viral proteins revealed that virus can be explored for drugs and drug carriers in biotech pharmacology and can be considered as valuable tools for drug delivery across membranes mainly in genetic therapy [39]. Moreover, viral-derived CPPs my replace viruses themselves as nanocarriers, showing advantage with respect to the potential biological hazards associated with viruses and the cost of the therapy [36, 40–42].

The presence of CPPs in viral proteins suggests that their function/requirement is conserved and optimized through evolution. Due the poor understanding of anionic CPPs class and the fact that they do not form a class by their own and they are assigned to different classes on a case-by-case basis, we cannot confirm that our NCC screened are genuine CPPs. An experimental validation of NCC as CPP is needed based on cell culture and murine models.

For the *de novo* motifs screened, no match was found against the Prosite collection of motifs. These results are expected; first, because these regions were defined as compositionally biased (CB) regions and per consequence were automatically filtered by the program. Secondly, the viruses are deeply under represented in these databases. Obviously, the (CB) can be a source of hypervariability providing a panel of antigenic variation like in HIV-1 and enhancing the capacity of virus to avoid host immune system [10].

## Conclusion

**Fig. 3:**
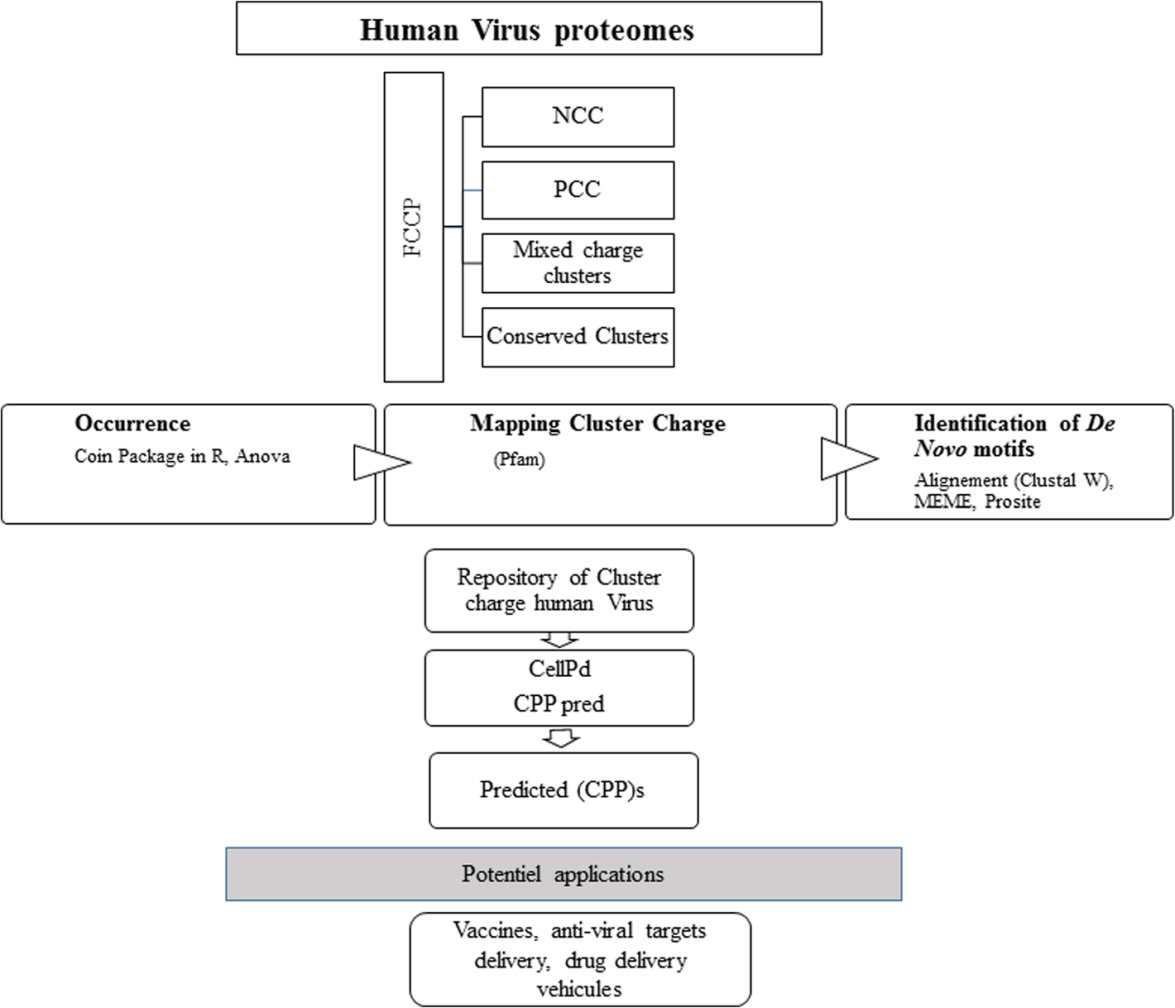
The main steps of screening Cluster Charge

The identification and the screening of clusters charges in proteins is a key analysis to asses any quantitative structure-function correlation in proteins. In this work, we tried to find for the detected linear clusters a function or role in protein interaction in viral proteomes (Fig. 3). Different approaches have been adopted to elucidate the multitude of viral structural proteins’ functions and to explain how they can co-evolve/interact with their hosts. Our suggested program provides a valuable tool to screen charge clusters in viral proteomes that may enrich our vision in determining the potential role of these clusters and enhance the research to better the current understanding of the molecular events driving virus transfections. Our tool has generated potential repository databases of anti-viral targets delivery and vaccines that can be explored for infectious diseases or treatment. In fact, this kind of targeting strategy can be used in cancer tumor, or on diagnosis and drug delivery vehicles that can locate cancer cells and helps treat or remove tumors [43].

## Methods

All viral proteins sequences were retrieved and downloaded in FASTA format from the Uniprot database (http://uniprot.org/proteomes/) [44] in order to reduce redundancy. One thousand and four hundred complete human virus proteomes, corresponding to 2549 unique proteins, were screened.

The FCCP algorithm was used, as previously described in the work of Belmabrouk et al. [24] to scan these protein sequences for the presence of charge clusters. The program parameters were set as follows: window size: 20; significance threshold level: 10^−5^. For more stringent filter conditions, we consider only CC starting and ending by a charged residue so at the end we can get CC shorter than 20 residues (see further details and recommendations on the use of the FCCP algorithm in Belmabrouk et al. [24].

The Pfam protein family database (http://pfam.xfam.org) [27] was downloaded and used in order to map CCs relative to functional domains within proteins.

Analysis of variance (ANOVA) was performed in order to test the difference between length and size of clusters between virus groups and families. The Trends chi-square test was used to evaluate the difference between the distributions of charge clusters according to their position relative to functional domains using functions of the *Coin* package in R language (http://www.r-project.org/). alignments were performed using Clustal *W*as implemented in MEGA program (version 4) [45].

To discover *denovo* motif, we used MEME (Mutiple EM for Motif Elicitation, version 4.10.2)(http://meme-suite.org/)]. These motifs were then compared against the PROSITE collection of motifs using the ScanProsite (http://prosite.expasy.org/scanprosite/) [33] was performed to related proteins.

## Additional files

Additional file 1: Virus Families of Positive Charge Clusters. % is (the number of Positive Charge Cluster present in each family/the total number of Positive Charge Clusters (*N* = 162)) *100; *N* = 162. (TIFF 49 kb)
Additional file 2: Virus group of Positive Charge Clusters. % is (the number of Positive Charge Cluster present in each group/the total number of Positive Charge Clusters (*N* = 162)) *100; *N* = 162. (TIFF 41 kb)
Additional file 3: Distribution of CC according to Pfam database: A. Distribution of PCC and NCC according to Pfam database: B. Distribution of mixed PCC and mixed NCC according to Pfam database: C. Distribution of Conserved PCC and NCC according to Pfam database. (TIFF 130 kb)
Additional file 4: Virus Families of Negative Charge Clusters. % is (the number of Negative Charge Cluster present in each family/the total number of Negative Charge Clusters (*N* = 211)) *100; *N* = 211. (TIFF 42 kb)
Additional file 5: Virus groups of Negative Charge Clusters. % is (the number of Negative Charge Cluster present in each group/the total number of Negative Charge Clusters (*N* = 211))*100; *N* = 211. (TIFF 25 kb)
Additional file 6: Virus Families of Mixed Charge Clusters. % of (the number of proteins which contained mixed Charge clusters in each family/total number of proteins contained mixed Charge cluster (*N* = 29)) *100; *N* = 29. (TIFF 27 kb)
Additional file 7: Distribution of conserved Charge Clusters in virus families. (TIFF 32 kb)
Additional file 8:
*De Novo* motifs screened within cluster charge. (TIF 44 kb)
Additional file 9: Results of Multiple Alignments: A. Result of Multiple Alignments of Positive Charge Clusters: B. Result of Multiple Alignments of Negative Charge Clusters. (ZIP 3 kb)

## Abbreviations

ANOVA: Analysis of variance
BCR: B-cell antigen receptor
CB: Compositionly biased
CC: Cluster charge
CellPPd: Designing of cell penetrating peptides
CPP: Cell penetrating pepetides
DNA: Deoxyribonucleic acid
dsDNA: Double-stranded DNA
dsRNA: Double-stranded RNA
FCCP: Finding cluster charge protein
HIV1: Human immunodeficiency virus
MEME: Mutiple EM for motif elicitation
NCC: Negative cluster charge
PCC: Positive cluster charge
RNA: Ribonucleicacid
ssDNA: Single stranded DNA
ssRNA: Single stranded RNA
