# Supplementary figures and images for: Screening for clusters of charge in human virus proteomes
Source: BMC Genomics. 2016 Oct 17;17(Suppl 9):758. doi: 10.1186/s12864-016-3086-3 (PMC5073957; doi:10.1186/s12864-016-3086-3)

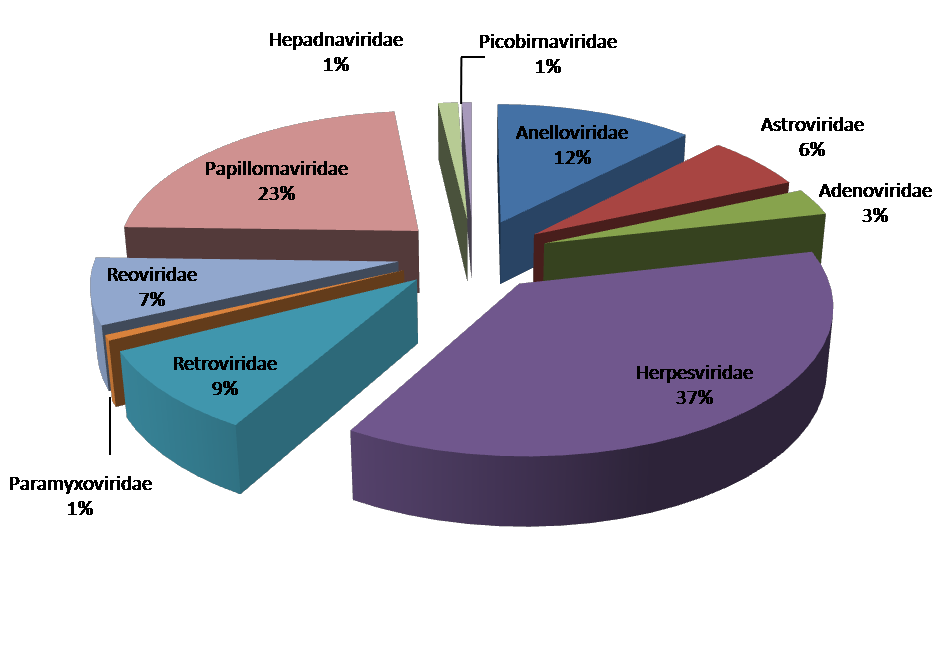

Supplement: Additional file 1: — Virus Families of Positive Charge Clusters. % is (the number of Positive Charge Cluster present in each family/the total number of Positive Charge Clusters (N = 162)) *100; N = 162. (TIFF 49 kb) [file 12864_2016_3086_MOESM1_ESM.tiff]

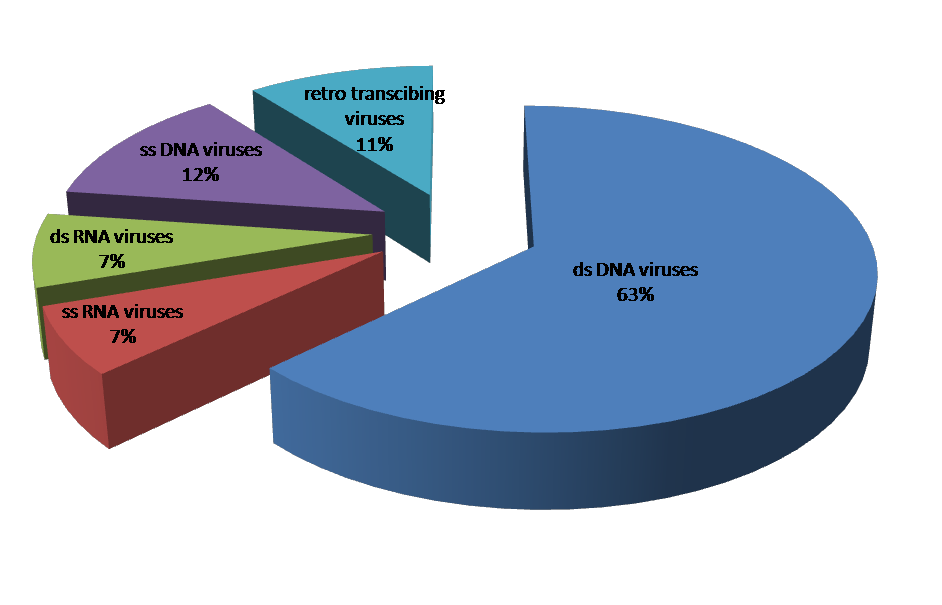

Supplement: Additional file 2: — Virus group of Positive Charge Clusters. % is (the number of Positive Charge Cluster present in each group/the total number of Positive Charge Clusters (N = 162)) *100; N = 162. (TIFF 41 kb) [file 12864_2016_3086_MOESM2_ESM.tiff]

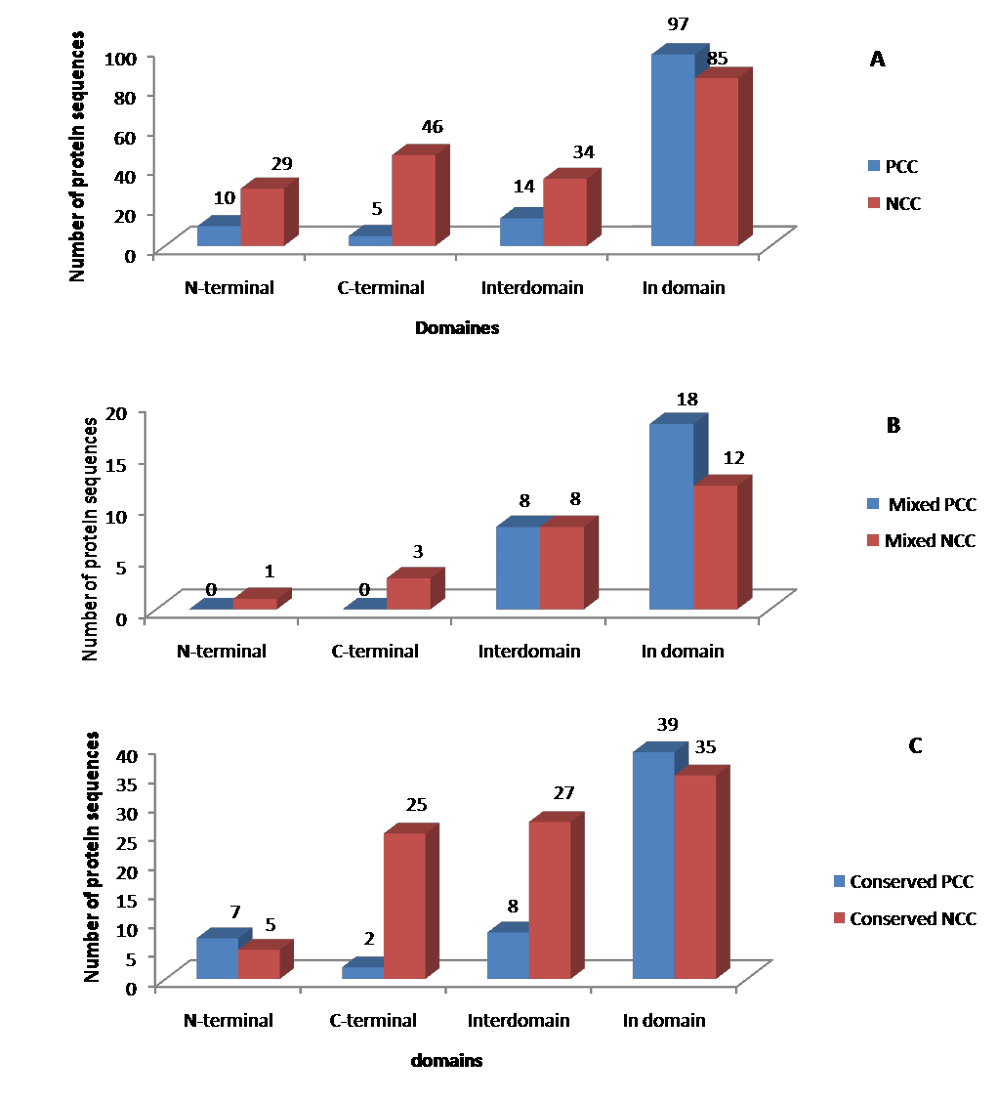

Supplement: Additional file 3: — Distribution of CC according to Pfam database: A. Distribution of PCC and NCC according to Pfam database: B. Distribution of mixed PCC and mixed NCC according to Pfam database: C. Distribution of Conserved PCC and NCC according to Pfam database. (TIFF 130 kb) [file 12864_2016_3086_MOESM3_ESM.tiff]

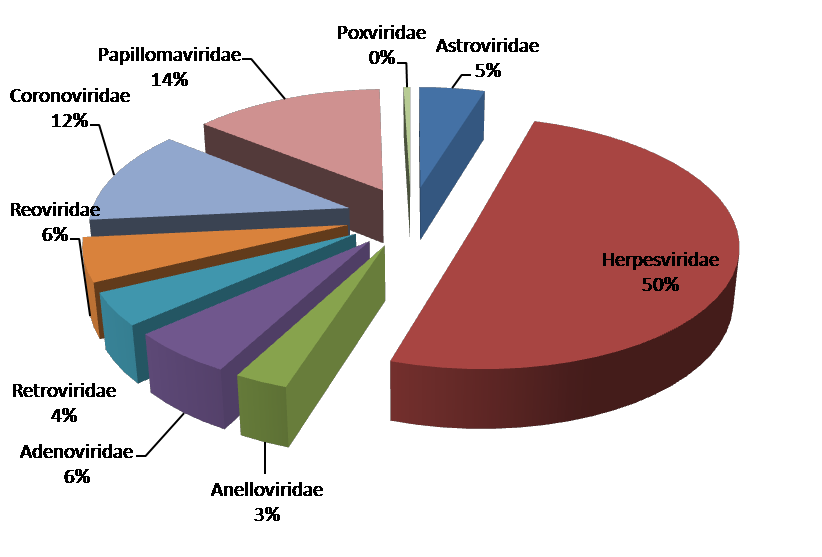

Supplement: Additional file 4: — Virus Families of Negative Charge Clusters. % is (the number of Negative Charge Cluster present in each family/the total number of Negative Charge Clusters (N = 211)) *100; N = 211. (TIFF 42 kb) [file 12864_2016_3086_MOESM4_ESM.tiff]

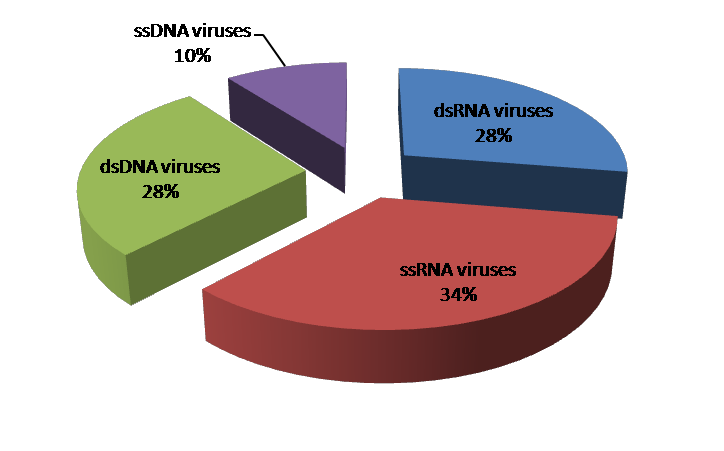

Supplement: Additional file 5: — Virus groups of Negative Charge Clusters. % is (the number of Negative Charge Cluster present in each group/the total number of Negative Charge Clusters (N = 211))*100; N = 211. (TIFF 25 kb) [file 12864_2016_3086_MOESM5_ESM.tiff]

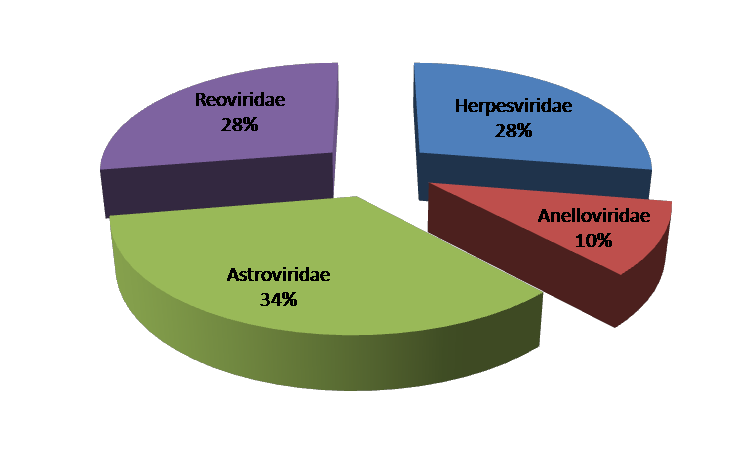

Supplement: Additional file 6: — Virus Families of Mixed Charge Clusters. % of (the number of proteins which contained mixed Charge clusters in each family/total number of proteins contained mixed Charge cluster (N = 29)) *100; N = 29. (TIFF 27 kb) [file 12864_2016_3086_MOESM6_ESM.tiff]

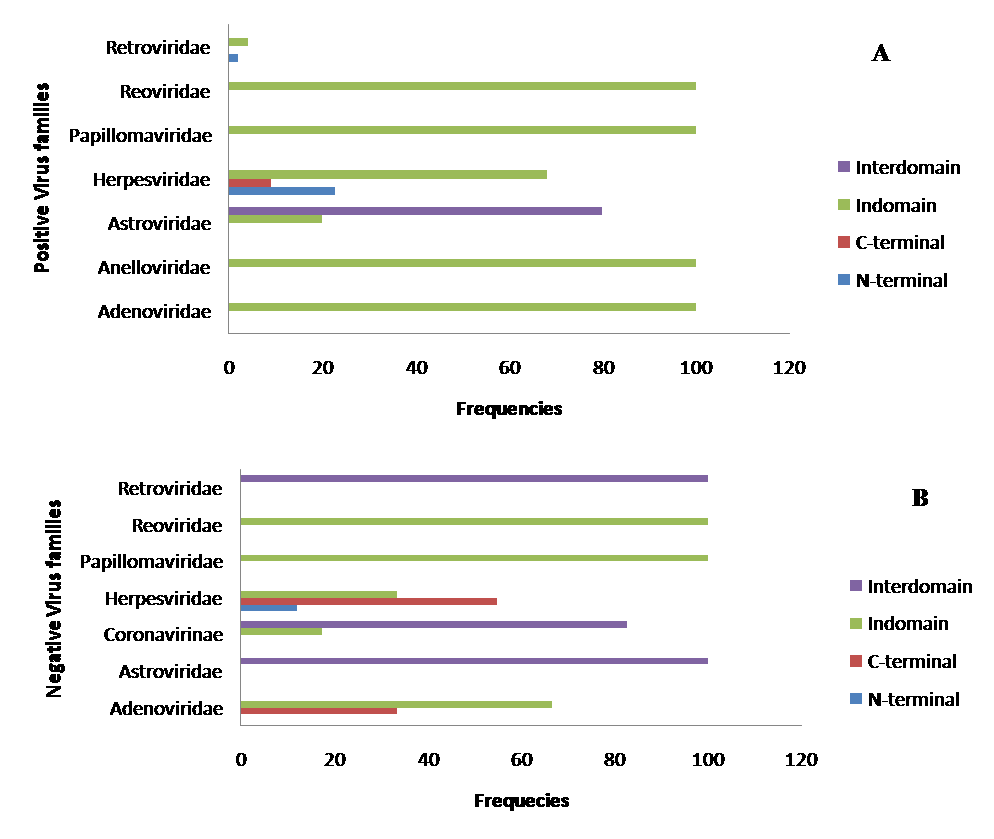

Supplement: Additional file 7: — Distribution of conserved Charge Clusters in virus families. (TIFF 32 kb) [file 12864_2016_3086_MOESM7_ESM.tiff]

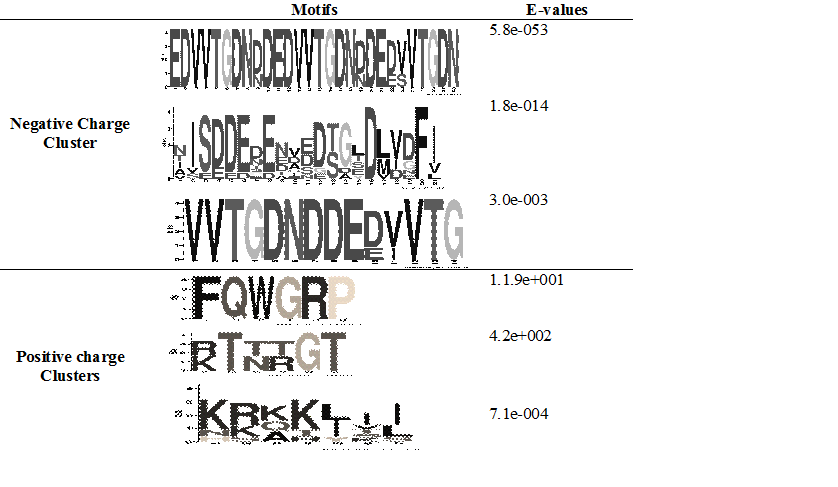

Supplement: Additional file 8: — De Novo motifs screened within cluster charge. (TIF 44 kb) [file 12864_2016_3086_MOESM8_ESM.tif]
